# Cultural Feasibility of Conversational Robots for Dementia Care in India: Participatory Design Study

**DOI:** 10.2196/80457

**Published:** 2025-11-06

**Authors:** Maria R Lima, Nivedhitha Srinivasan, Sarah Daniels, Sridhar Vaitheswaran, Ravi Vaidyanathan

**Affiliations:** 1 Department of Mechanical Engineering Imperial College London London United Kingdom; 2 Care Research and Technology Centre UK Dementia Research Institute London United Kingdom; 3 Dementia Care Schizophrenia Research Foundation India Chennai India

**Keywords:** conversational artificial intelligence, socially assistive robots, dementia care, low- and middle-income countries, artificial intelligence

## Abstract

**Background:**

Dementia poses a significant challenge in India. The rising incidence rates, limited resources, and restricted clinician access have contributed to a staggering 90% gap in diagnosis and care. Conversational technology provides a natural user interface with the potential to promote the independence, well-being, and safety of people living with dementia at home. However, the feasibility of implementing such technology to support people living with dementia across diverse cultural and economic settings remains underexplored.

**Objective:**

This study aimed to assess the cultural feasibility of conversational robots for dementia care in India, a culturally underserved context in robotics and artificial intelligence (AI) for aging and dementia care.

**Methods:**

We involved 29 stakeholders, including people living with dementia, caregivers, and dementia care professionals. We evaluated (1) the engagement of people living with dementia with 3 conversational robots with varying interactive modalities (a voice agent, a virtual affective robot, and an embodied robot), (2) robot acceptance, and (3) stakeholder perspectives on the benefits and challenges of deploying conversational AI in India.

**Results:**

People living with dementia were willing to engage in verbal dialogue with conversational robots. Stakeholders perceived the technology as beneficial for supporting daily tasks at home, reducing loneliness, and enhancing cognitive function. We identified design adaptations to address feasibility challenges in India, including the need to (1) adapt interaction style to use a kind tone, appreciative language, and customizable facial expressions; (2) improve speech recognition for local accents interpretation and noisy settings; and (3) introduce prototypes in local clinics to promote familiarity.

**Conclusions:**

This work offers novel insights into cultural acceptance, human-robot engagement, and perceived utility for dementia care, along with key design implications for integrating conversational AI into care settings in India.

## Introduction

### Background

Dementia is a growing concern worldwide as the population ages. By 2050, the aging demographic is projected to double, with the number of people living with dementia anticipated to reach 139 million [1]. This surge is particularly pronounced in low- and middle-income countries, where approximately 63% of people living with dementia live [2]. The challenge of dementia in India stands out, given its vast population and rising incidence of dementia, coupled with significant gaps in resources and clinician accessibility. Today in India, 8.8 million people aged 60 years and older live with dementia, a figure that is projected to exceed 14 million by 2050 [3]. Yet, the diagnosis, care, and treatment landscape in India reveals a staggering 90% gap [4]. Addressing the challenge of dementia in India requires an understanding of the sociocultural factors that limit access to care and a widespread lack of dementia awareness among all stakeholders. Studies in India indicate that people living with dementia can be stigmatized and neglected by society and their families, creating critical barriers to accessing care [5]. Furthermore, the financial and mental strain placed on caregivers and family members is enormous [6]. Research in South India underscores the demand for stronger technology adoption to support people living with dementia and caregivers [7].

### Conversational Artificial Intelligence for Health and Well-Being in Older Age

Socially assistive robots (SARs) and conversational artificial intelligence (AI) technologies hold potential to enhance independence and well-being at home [8-10]. Examples of home assistance include medication management and reminders [11], cognitive stimulation and engagement [12], companionship [13], and activities of daily living [14], while relieving the burden placed on caregivers [15]. Furthermore, SARs have shown potential in improving neuropsychiatric symptoms [16] and reducing agitation [17].

Voice interaction provides an accessible and natural user interface to interact with technology without the need for complex controls, promoting usability among older adults [18] and providing safety in scenarios where voice may be the only form of communication. For instance, conversational AI integrated with home monitoring technology can track behavior and alert stakeholders to risks, such as falls or cognitive decline [19]. Conversational SARs offer multimodal interactive features beyond speech, including facial expressions, embodiment, and gestures. Previous work has argued that physical embodiment and social presence can enhance engagement and enjoyment in human-robot interaction (HRI) [20-22]. Tapus et al [23] defined engagement as a sustained collaborative connection between humans and robots. Heerink et al [24] reported social presence as a key robot design feature for older people, while Nishio et al [22] showed that older adults were more verbally engaged with an embodied robot than a virtual one. Despite the promise of conversational robots, the effectiveness in supporting well-being and daily living activities among people living with dementia remains underexplored.

### Cultural Adaptation

User-centered design with stakeholders is critical for developing useful and acceptable robots [25]. This approach can help identify perceived benefits and barriers for specific support contexts during the early stages of HRI design [26]. Previous research suggests that cultural factors influence robot acceptance [27,28], making it crucial to consider these in robot design. However, few studies in robotics for dementia care have involved stakeholders [29-33], and none of these have addressed the necessary cultural adaptations and feasibility challenges inhibiting real-world deployments in low- and middle-income countries, particularly in India.

While there is a lack of studies exploring SARs for dementia care in India, their acceptance in supporting daily care and well-being is growing among Indian older adults [34,35]. To the best of our knowledge, this is the first study to investigate the cultural feasibility of conversational SARs for dementia care in India through stakeholder involvement. In the context of this study, cultural feasibility refers to the suitability and acceptance of technology to support dementia care within India’s cultural context, including stakeholders’ perceptions and the barriers to its integration into daily life. This is particularly pertinent in India, given sociocultural factors that shape user perceptions of robots for well-being support in residential settings, such as deep-rooted family caregiving responsibilities and varying levels of digital literacy across rural and urban populations [36].

### Research Questions

In this study, we assessed the cultural feasibility of conversational robots in India. We engaged a total of 29 stakeholders, including people living with dementia, caregivers, family members, and dementia care professionals. People living with dementia interacted with 3 conversational robots with varying interactive modalities. We evaluated engagement through behavioral observations and video analysis, assessed robot acceptance using standardized questionnaires, and gathered stakeholders’ attitudes and perceptions through interviews. Our research was guided by the following research questions (RQs):

- RQ1. Do people affected by dementia in India engage in verbal interactions with conversational robots, and how do different interactive modalities affect user engagement?
- RQ2. Do stakeholders accept the use of conversational robots in India?
- RQ3. What feasibility challenges need to be addressed for effective integration of conversational robots for dementia care in India?

## Methods

### Study Design

We engaged a total of 29 stakeholders in India, including people living with dementia (n=11), formal and informal caregivers (n=11), and dementia care professionals (n=7). People living with dementia, often accompanied by a caregiver or both a caregiver and a family member, participated in a single session that involved verbal dialogue with 3 conversational robots. Each session lasted approximately 45 minutes and included a semistructured interview. We encouraged caregivers and dementia care professionals who observed the sessions to share their perceived benefits and barriers to using technology for dementia care in India.

**Figure 1:**
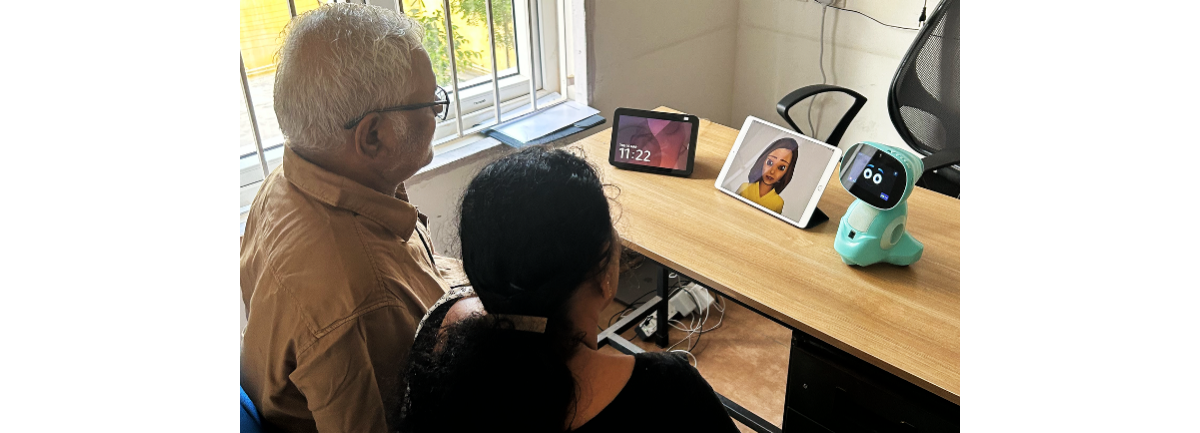
Experimental setup with a participant and caregiver facing the robots.

After providing informed consent, learning about the purpose of the study, and having the opportunity to ask questions, participants verbally engaged with the robots in English. Robots were placed on a table in front of participants (Figure 1), one at a time. We aimed to understand how people perceived robots with different interactive modalities: (1) voice only, (2) facial expressions with voice, and (3) embodiment combined with voice and facial display. Accordingly, robots were presented to participants in the same order to minimize distractions. A psychologist and a researcher jointly assessed engagement and moderated the session, respectively, while a video camera recorded the session. Data were collected via direct observation of engagement, video recordings for offline assessment of engagement, a robot acceptance questionnaire, and a semistructured interview to explore the perceived benefits and barriers to technology adoption in India.

### Participants and Setting

We involved 11 people living with mild to moderate dementia, as determined by established clinical assessments (mean age 78.6, SD 8.95; 2 female participants). Two weeks before the study, participants’ cognitive and daily function were evaluated using the Alzheimer’s Disease Assessment Scale-Cognitive Subscale (ADAS-Cog) [37] and the Clinical Dementia Rating (CDR) [38] (mean ADAS-Cog 21.4, SD 7.1; mean CDR 1.8, SD 0.5). Participants were recruited based on the severity of cognitive impairment and communication ability. Specifically, we included participants with a CDR score of ≤2 (mild to moderate cognitive impairment) and sufficient ability to engage verbally in English with the robots. The study excluded individuals who were unable to provide informed consent or had impairments that precluded them from hearing or seeing the robot.

The study was conducted at the Schizophrenia Research Foundation (SCARF) Centre for Active Ageing and the in-patient unit of the Sri T S Santhanam Centre for Elderly Care in Chennai, India. All participants lived in urban settings and had an educational background (mean years of education 13.2, SD 4.0). Most participants (n=7) lived at home with their partner, 2 had formal caregivers, and 2 were inpatients. Participant information is detailed in Table S1 in Multimedia Appendix 1. Generally, participants were not familiar with voice technologies. Of the 3 participants who had heard of commercial devices, including Amazon Alexa, none had used them.

### Conversational Robots

Participants engaged with 3 conversational social robots (Rs): a smart speaker (R1), a virtual affective robot (R2), and an embodied social robot (R3). R1, an Amazon Echo device, prompted users with a well-being questionnaire querying mood, sleep, fatigue, anxiety, activity plans for the day, and a breakfast recall to trigger memory of a recent event. The well-being questionnaire was developed following our previous work that analyzed daily Alexa interactions in households with people living with dementia [19]. R1 interacted through speech and screen displays. Participants initiated the questionnaire using a specific invocation phrase, as instructed by the researcher.

R2, a research platform built on our previous work [39], is a virtual affective robot displayed on a tablet that simulates facial expressions. The robot’s face was integrated with a bespoke conversational agent designed to assist in daily activities, offering support based on the user’s blood pressure readings. R2 interacted through speech combined with a virtual affective face.

R3, the social robot Miko, displayed facial expressions through animated eyes and mouth [40] and included physical embodiment. While R3 could move autonomously, its motion was restricted for this study to avoid distracting behaviors. Participants initiated dialogue with R3 using an invocation name, as instructed by the researcher.

Interactions with R1 and R2 followed a structured, health-related questionnaire. R1 interacted through voice and screen display, while R2 combined voice with a virtual affective face, simulating basic expressions. In both cases, the robots adapted their verbal responses based on user input (eg, the robot asked for details if the user expressed concern). In contrast, interactions with R3 were more open-ended and social: participants asked general questions about the robot (eg, “Hello Miko, where are you from?”) and culturally specific questions (eg, “Hello Miko, what is famous in Chennai?”) from a provided list and were encouraged to ask additional questions of personal interest. Interactions with R3 had a higher degree of multimodality, combining voice with the robot’s physical embodiment and animated facial expressions. Average dialogues with R1, R2, and R3 lasted 4 minutes 53 seconds, 4 minutes 3 seconds, and 4 minutes 52 seconds, respectively. Examples of interaction transcripts are provided in Multimedia Appendix 1.

### Data Collection

#### Engagement Assessed On-Site

During the sessions, a trained psychologist recorded user engagement using a modified version of the Observational Measure of Engagement (OME) [41]. We considered 5 dimensions: attention and attitude to stimulus, both rated on a scale from 1 (very negative) to 7 (very positive), based on the average level during interactions; cognitive difficulty with the task, rated from 1 (easy to interact) to 5 (very challenging); and frequency of disruptions or distractions, rated from 0 (none) to 3 (during most or all of the session). Details of OME measures are provided in Table S2 in Multimedia Appendix 1.

#### Engagement Assessed From Video Analysis

We used a video coding protocol [42] to evaluate participants’ emotional, visual, and verbal engagement with the robots. This method has been previously applied to evaluate the engagement of people living with dementia interacting with SARs [43]. Engagement was calculated as the percentage of total interaction time during which a participant exhibited specific behaviors. Two researchers independently analyzed each video recording (Cohen κ=0.71, indicating good agreement).

We examined the duration of positive (eg, smiling and greeting the robot), negative (eg, showing anger, fear, and sadness), or neutral emotional engagement. Visual engagement included being engaged with the robot (eg, maintaining eye contact), the facilitator, or not engaged. We further measured the duration of positive (eg, actively responding to the robot’s statements) or negative (eg, limited participation and repetitive answers) verbal engagement in HRIs. Engagement metrics from video analysis are provided in Table S3 in Multimedia Appendix 1.

#### Robot Acceptance and Cultural Feasibility

We assessed acceptance of each robot using a questionnaire tailored for older adults’ care robots [44]. The 11-item questionnaire evaluates attitudes toward robots across 5 constructs: sociability, enjoyment, usefulness as an assistive tool, feelings of anxiety when using the robot, and trust in the robots’ advice, each rated on a 5-point Likert scale (refer to Table S4 in Multimedia Appendix 1). Participants shared their views on the 3 robots in a semistructured interview. We explored stakeholders’ preferences for robot interaction styles, as well as perceived benefits and barriers within the cultural context of India, including insights from people living with dementia, caregivers, family members, and dementia care professionals.

We conducted a thematic analysis following the steps outlined by Braun and Clarke [45] to identify recurring themes that reflected stakeholders’ attitudes toward using conversational robots for dementia care in India. Following an inductive approach, 2 coders independently reviewed interview transcripts, developed a preliminary list of codes, and then met to discuss their individual findings and identify a structured set of themes and subthemes.

### Ethical Considerations

The study was approved by the Ethics Committee of SCARF, India (Protocol SRF-DC/17-A1/JUN/2021). Written informed consent was obtained from all participants before participation. Participants were informed that sessions would be video recorded for research purposes, that only deidentified information would be analyzed, and that data were securely stored on local institutional servers and were accessible only to members of the research team. Participants were compensated with INR 1000 (US $11) for their time in the session. They retained the right to withdraw from the study at any time.

## Results

### Overview

We first present findings from engagement with the robots, assessed both from on-site behavior observation and video analysis. We further report results from robot acceptance questionnaires and insights from semistructured interviews.

### Human-Robot Engagement

#### Behavior Observation

Of the 33 HRIs (since 3 robots were tested by each of the 11 people living with dementia in our stakeholder group) 1 session was excluded from analysis due to software issues with R2. Using the OME method, we compared 5 engagement metrics observed by a trained psychologist for each robot. As shown in Table 1, attention and attitude were generally positive across all robots. Higher mean scores for attention and attitude were observed for R2 (A1: mean 5.7, SD 0.8; A2: mean 5.3, SD 1.1; 7-point scale), followed by R3 (A1: mean 5.6, SD 0.9; A2: mean 5.2, SD 0.9) and R1 (A1: mean 5.1, SD 0.9; A2: mean 5.0, SD 1.0).

**Table 1:** Engagement results expressed as mean (SD) using metrics: attention (A1), attitude (A2), cognitive difficulty (CD), frequency of disruptions (D1), and distractions (D2) for the 3 conversational robots tested: a smart speaker (R1), a virtual affective robot (R2), and an embodied social robot (R3).

Metric	Scale	R1, mean (SD)	R2, mean (SD)	R3, mean (SD)
A1	1-7	5.1 (0.9)	5.7 (0.8)	5.6 (0.9)
A2	1-7	5.0 (1.0)	5.3 (1.1)	5.2 (0.9)
CD	1-5	1.7 (1.0)	1.8 (1.1)	2.2 (1.3)
D1	0-3	0.3 (0.7)	0.3 (0.7)	0.3 (0.5)
D2	0-3	0.4 (0.7)	0.4 (0.7)	0.2 (0.4)

Verbal interactions with R3 were, on average, more cognitively challenging (cognitive difficulty [CD]: mean 2.2, SD 1.3; 5-point scale) compared to the other 2 robots (R2: CD mean 1.8, SD 1.1; R1: CD mean 1.7, SD 1.0). We observed increased difficulty interacting with the embodied robot R3 due to the need to recall its invocation name for each query, which often resulted in participants asking R3 a question without activating its listening mode. The other OME dimensions were rated consistently for the 3 robots, and no significant differences were found in the dimensions analyzed.

#### Video Analysis

**Figure 2:**
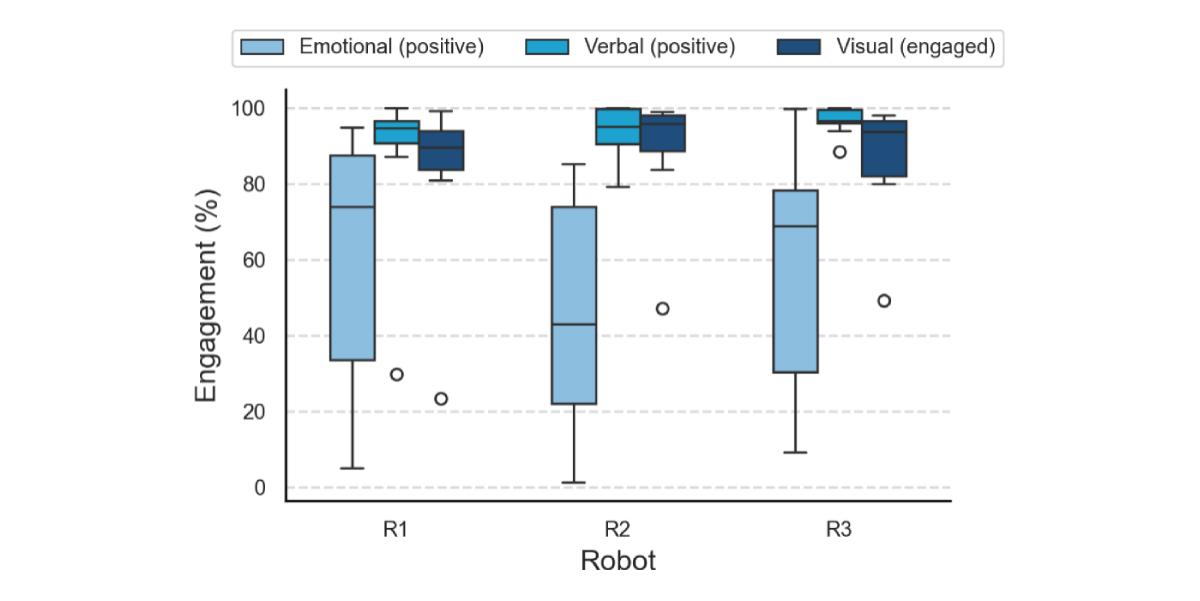
Engagement assessed from video analysis (percentage of total interaction time). Selected metrics showing higher variations across robots are shown: positive emotional, positive verbal, and visual engagement. R: conversational social robot.

We evaluated emotional (positive, neutral, and negative), verbal (positive and negative), and visual (engaged with the robot, engaged with the facilitator, and not engaged) engagement from the video recordings. Figure 2 shows the distribution of the selected engagement metrics across the cohort for each robot condition (R1, R2, and R3). While no significant differences were reported, variations and trends were observed at the cohort level. Values represent the percentage of the total interaction time. Positive emotional engagement showed the greatest variability, with R1 having the highest median (73.9%, IQR 53.9%), followed by R3 (68.9%, IQR 48%) and R2 (43%, IQR 51.8%). Participants exhibited less positive and more neutral emotional responses with R2. Given the high OME attention scores, this suggests that while participants were generally attentive to R2, they did not display positive reactions when interacting with the affective virtual face.

Verbal engagement with all 3 robots remained high, with median positive verbal engagement exceeding 94% of the total interaction time. Participants were generally visually engaged with the robots, maintaining attention and eye contact. Median values exceeded 89% of total interaction time, with R1 eliciting slightly lower visual engagement overall. These findings showed that participants positively engaged with all 3 conversational robots in verbal interactions, with no significant differences in engagement based on robot interaction modalities across the cohort.

### Robot Acceptance

People living with dementia (n=11) and caregivers (n=11) completed a posttrial questionnaire to assess their robot acceptance, overall experience, and attitudes toward adopting conversational robots in India. Caregivers responded based on their observations and how they envisioned the technology in their care setting.

**Figure 3:**
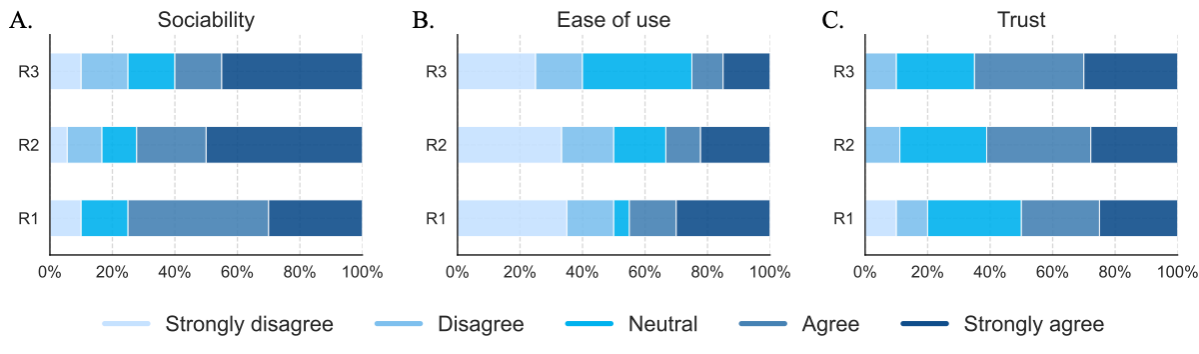
Distribution of participant perceptions by robot condition for (1) sociability, (2) ease of use, and (3) trust. R: conversational social robot.

Table 2 presents descriptive statistics for each robot condition and the reliability coefficient (Cronbach α) of the underlying constructs. Although no statistically significant differences were reported between the means of each construct, some trends emerged. In general, participants expressed positive attitudes toward the conversational robots. Perceived sociability and usefulness had the highest mean values, indicating that participants viewed the robots as useful tools to engage in verbal dialogue and support well-being and independence at home. Particularly, 80% of respondents agreed that Alexa (R1) could improve social interaction skills, 78% agreed with the virtual affective robot (R2), and 65% with the embodied robot (R3; Figure 3A).

**Table 2:** Results expressed as mean (SD) of robot acceptance across 5 constructs rated on a 5-point Likert scale. Cronbach α was computed as a reliability measure of the underlying constructs. The anxiety construct is reverse-scored.

Construct	R1, mean (SD)	R2, mean (SD)	R3, mean (SD)	Cronbach α
Sociability	3.9 (1.1)	4.1 (1.2)	3.8 (1.4)	1
Ease of use	3.0 (1.8)	2.9 (1.6)	3.0 (1.5)	1
Usefulness	3.9 (1.1)	4.1 (1.1)	3.9 (1.0)	1
Anxiety	1.9 (1.3)	1.8 (1.3)	1.8 (1.4)	0.9
Trust	3.5 (0.9)	3.7 (1.0)	3.7 (1.0)	0.8

Lower mean values were generally found in the perceived ease of use (Table 2), as participants noted they were not ready to use such systems independently due to unfamiliarity with voice technologies. Approximately 50% of respondents felt they could not interact independently with R1 or R2, and 40% reported the same for R3 (Figure 3B). The anxiety subdimension (reverse-scored in Table 2) showed low mean values across all robots, indicating that people living with dementia and caregivers did not find the robots intimidating. Furthermore, the majority expressed trust in following the robots’ suggestions or action prompts, with 65% of respondents agreeing for R3, 61% for R2, and 50% for R1 (Figure 3C).

### Perceived Utility for Dementia Care in India

We explored stakeholders’ perceived benefits and barriers of using SARs for dementia care in India. Views were collected from the full stakeholder group, including people living with dementia, formal and informal caregivers, and dementia care professionals, providing expert-informed insights into how this technology could work in the person’s daily routine. Three main themes emerged from the interviews: (1) perceived valuable support scenarios to promote dementia care in home and clinical settings in India, (2) preferences for robot interactive modalities, and (3) factors affecting feasibility in India.

### Support Scenarios

#### Support Routine at Home

One of the most widely reported benefits of conversational technology was the ability to learn and support routines at home. Emphasis was placed on providing personalized reminders for daily tasks, including medication and medical appointments, and monitoring daily activities as people become more forgetful: “I tend to be forgetful; it would be good to have a robot that remembers what I have to do and reminds me at the appropriate time” (P3). In addition, robots proved beneficial in monitoring behavior and alerting caregivers to agitation, restlessness, wandering, or falls: “It could inform someone where I am if I fall” (P3), while offering verbal support to the user during such events: “It is more voice to give advice” (C1). The kitchen was particularly mentioned as a place where a robot could check and alert users of appliances left turned on. However, participants expressed reservations about the potential intrusiveness of adding monitoring devices at home and their associated costs.

#### Help With Loneliness

Participants remarked that conversational AI could help alleviate loneliness by providing companionship, entertainment, or answering repetitive questions: “...he is alone most times and would feel like somebody is talking to him” (C4). This was noted to ease the load on caregivers: “because he is always lonely, and I am always busy [a conversational robot] can help in our daily life” (C4). It was further suggested that having a robot at home could foster family connections: “I could bring the grandchildren and connect better with them” (P5), and support daily motivation: “[the robot] could give a positive note in the morning to start the day in a good mood” (C7).

#### Enhance Cognitive Function

Participants highlighted the potential for HRI as a means of enhancing cognitive engagement and communication skills: “His cognition may improve if he holds a conversation every day” (C6). Dementia care professionals indicated that this would be beneficial for people living with early to moderate dementia who can verbally communicate with the robot and suggested that, if well accepted by people living with dementia and used consistently, the robot could detect early signs of cognitive decline through language use or shifts in emotional state from the tone of voice and inform the caregiver promptly. Examples mentioned by participants include the risk of delusions, hallucinations, and suspicious thoughts. They also expressed that robots should be used carefully when dealing with people with behavioral problems such as psychosis, as these individuals may misinterpret the robot’s voice as a hallucination: “...they may end up thinking it’s a real person” (D1). Participants highlighted the importance of avoiding emotional deception with the use of SARs, which has been raised as an ethical concern in previous studies [46].

### Robot Design Preferences

#### Personalized Interactions

We found that the people living with dementia tend to trust a robot more when they find it useful, irrespective of its design and interactive modalities. One caregiver noted: “If he understands it is useful for him, then he will pick it up quickly and want to use it” (C6). Caregivers and dementia care professionals advocated for personalization of interactions and support to the individual’s needs over time. This may include considerations of their medical history, dementia progression, and their comfort with the robot. As one participant emphasized: “[The robot needs to] know more about me to give reliable advice” (P3), highlighting the importance of personalization to provide meaningful support. Furthermore, the ability to adjust the robot’s speech rate and response time emerged as a key design feature, allowing for tailored interactions to individual cognitive needs. The need for personalization as a key feature, as voiced by stakeholders, is aligned with previous studies [30,32].

#### Robot Appearance

Participants remarked that the robot’s appearance should be culturally appropriate. This includes the robot’s facial expressions, tone of voice, and choice of words. We observed that R2’s affective face generally facilitated initial interest among participants; however, overall positive emotional engagement was generally lower compared to the other robots (Figure 2). Interviews uncovered mixed perceptions about adding an affective face to robots. While some thought it enhanced engagement and retained attention: “having a face is always good, so I can connect, so I don’t have the feeling of talking to a device” (P8), others were wary of its human-like features and prioritized the practical assistance offered: “just voice is enough...Alexa serves the purpose” (C4). Stakeholders suggested having the option to culturally customize the robot’s appearance to make it more relatable, including its eye colors, adding a bindi, and modifying clothes.

#### Task-Based Versus Conversational Style

Discussions uncovered mixed views on the preferred communication style. While some favored a direct, task-based approach to assist with specific activities or needs: for example, “I like straightforward, I ask a question, the robot answers” (P7), others valued the ability to engage in open dialogue as a conversational partner about personalized topics of interest. As one caregiver noted: “...it could be like a ChatGPT that he could hold a conversation with every day” (C6). We found that these views varied with the cognitive abilities of the user in question, with task-based interactions generally favored for individuals with worse cognitive impairment. This preference aligns with previous participatory research highlighting concerns related to the use of open-ended questions that may generate anxiety among people living with dementia [26]. Overall, stakeholders preferred the robot to communicate in a kind and appreciative way, maintaining user comfort and reflecting cultural values: “[Robots] need more appreciative comments so the elderly feel encouraged to talk” (C2).

#### Challenges for Use in India

While participants recognized the promise of conversational AI in their dementia care context and were generally open to its future use, they underscored the need for improvements before real-world deployments in home and clinical settings in India.

#### Verbal Communication

Participants remarked on the need for clarity in speech and local accent interpretation for effective communication with target users: “[The robot] has to understand me, the way I speak” (P1). The ability to speak in local Indian languages was not deemed essential. However, participants expressed preferences for a robot that communicates in an Indian English accent, which, they noted, would increase both technology acceptance and ease of human-robot verbal interaction. In fact, participants generally favored a robotic platform based on speech comprehension: “I prefer this robot because I can better understand what it says” (P6). Furthermore, speech recognition emerged as a critical area for improvement. Such errors often led to conversation restarts or repeated robot answers. Background noise derived from fans often caused delays in speech processing. For effective communication, the ability to process longer speech, pauses, and handle background noise should be incorporated, particularly in the Indian setting, which is characteristically noisy both indoors and outdoors. During HRI sessions, we observed that participants’ attitudes often changed when the robot misinterpreted their speech, sometimes leading them to repeat the instruction with an aggressive tone. Interestingly, one participant empathized with the robot’s speech recognition limitations: “Alexa, you are not able to follow me, ma’am? ...If you need any help, I’ll tell you” (P11).

#### Familiarity and Accessibility

Participants stressed that the current older generation in India would be hesitant to accept and adopt conversational robots due to unfamiliarity. As one caregiver noted: “she is amazed by these devices but will need help to learn how to use them” (C8). However, this barrier was expected to diminish as future generations become more accustomed to voice technology. Emphasis was placed on a phased introduction with a training period to build trust and familiarity: starting with robot prototypes in clinical settings, followed by a recommendation from a health care professional to use it, and finally integrating it into the household. The importance of familiarity in shaping willingness to engage with the technology was also emphasized: “I would need to know how [the robot] can contribute to me, what I can teach them, and what I can learn from them to know whether I would like to interact with it” (P3).

We found that the robot’s design should prioritize ease of use, ensuring people living with dementia are not overwhelmed by its features: “...he needs to be familiar with [the technology], I don’t want to add anything more complex” (C1). Participants further suggested that the robot should be well integrated into the household to promote familiarity and comfort among people living with dementia, making them feel the technology is “among the family, instead of being designed just for him” (C7). In addition, culture-focused discussions revealed concerns about the affordability of the technology: “These conversational robots are definitely useful, but are also expensive for lower-income classes to get them*”* (C7), and they rely on stable Wi-Fi. Conversely, there was a shared concern that if the robot malfunctions, people living with dementia would feel lost and lonely, highlighting that caregivers should be involved in the training phase to handle potential technical issues.

#### Privacy Concerns

Caregivers voiced concerns regarding continuous data collection from people living with dementia, particularly voice recordings and activity monitoring at home. They stressed the importance of transparency in data protection and sharing, with reservations about sharing medical details without clarity on who has access and how the data is used for patient benefit: “I don’t want to share what medication my mom is taking if I don’t know how the data is being handled, for that I am scared” (C8). In addition, while recognizing the potential of the technology to enhance and track cognitive skills, participants noted that the frequency of verbal interactions, the cultural sensitivity of conversations, and the robot’s communication style should be designed in a nonintrusive and culturally sensitive way to maintain user comfort and trust.

## Discussion

### Overview

Health inequality significantly increases the impact of dementia in underprivileged and lower socioeconomic communities [47,48]. The challenge of dementia in India stands out due to its vast population, high incidence rates, limited resources, poor clinician accessibility, and a striking 90% gap in diagnosis and care [4]. To address this gap, we involved 29 stakeholders, including people with lived experience of dementia, to investigate the cultural feasibility of conversational SARs to support dementia care in India. People living with dementia interacted with 3 robot platforms with different interactive modalities: a smart speaker (R1), a virtual affective robot (R2), and an embodied social robot (R3). Our study provides insight into (1) the engagement of people living with dementia with the conversational robots, (2) robot acceptance, (3) the perceived utility in dementia care, and (4) the challenges that need to be addressed for integrating this technology into real-world care settings in India.

### Principal Findings

Our findings indicate that people living with dementia positively engaged with the 3 robots in verbal dialogue, with variations in some engagement metrics (refer to RQ1). Participants exhibited shorter visual engagement durations and lower attention scores with R1, the smart speaker. Conversely, the perceived trust in this robot as an assistive companion was generally lower compared to the other platforms. However, it registered longer positive emotional engagement, which could be attributed to the novelty effect [49], that is, initial engagement that wears off over time, resulting in decreased interest and changing attitudes toward robots. Sessions were designed with an increasing level of multimodality in mind, allowing participants to experience the incremental interactive features each robot offered. For this reason, R1 was the first robot that participants interacted with, and given their overall unfamiliarity with voice technology, this suggests R1 sparked initial curiosity. However, the smart speaker R1 may lack interactive features to sustain attention over time compared to the other 2 robot platforms.

In addition, we found that overall R3 was more challenging to interact with, a finding supported by the robot’s lower ratings for its perceived role as a conversational partner. This difficulty may be attributed to R3’s requirement to be called by its invocation name for each query, which many participants found challenging to recall. Consequently, participants would often ask R3 a question without activating its listening mode. Despite these challenges in triggering the robot, R3 elicited high verbal engagement overall, with some participants independently asking questions beyond those on the list provided. This could be due to its multimodal interactive features, including embodiment. Research has shown that the physical embodiment and social presence of SARs can promote engagement compared with virtual agents [20,22,24,50]. Despite the overall engagement with R3, some participants perceived R3 as a “toy” and voiced concerns about feeling infantilized by technology.

Stakeholders were open to the adoption of conversational technology to support dementia care in India and identified key support functions (refer to RQ2). Supporting routines at home was viewed as a promising way for conversational AI to enhance the independence of people living with dementia while reducing the burden placed on caregivers. Emphasis was placed on reminders for daily tasks and alerts for behavioral risks, such as agitation or wandering. In addition, the potential to have regular conversations about topics of interest and prompt users to act in their daily routines was deemed beneficial to promote cognitive function and provide companionship.

Overall, our findings underscore the need to personalize verbal interactions to users’ cognitive needs over time, culturally adapt the robot’s communication style to sustain user comfort in task-based or more conversational tasks, and prioritize effectiveness in natural language communication. Building on these insights, the following section outlines design implications and recommendations for integrating conversational AI into dementia care settings in India.

### Implications

Our study identified improvements needed to culturally adapt conversational robots for dementia care in India (refer to RQ3). Our findings suggest improvements in speech processing in noisy environments and the ability to handle user pauses and longer speech. Response delays and turn-taking during verbal dialogue with robots are well-recognized challenges that remain unresolved [51]. Local accent interpretation should also be addressed. In our experiments, speech recognition errors often led to participant disengagement. The limited performance of automated speech recognition systems on Indian accents remains a recognized gap, largely due to the lack of representative training data [52]. However, recent research has shown promising improvements in both speech interpretation and generation of Indian-accented English. For instance, fine-tuning Whisper on Indian-accented speech has improved recognition accuracy, reducing the word error rate to 15% [53]. In addition, some commercial text-to-speech services provide Indian English voices, such as Amazon Polly, Microsoft Azure Neural TTS, and ElevenLabs. Future HRI feasibility studies in India could integrate these adapted speech recognition and synthesis models in conversational robotic systems to enhance user engagement.

Adapting the robot’s interaction style and appearance to align with cultural norms is another important aspect. Particularly, participants valued a communication style characterized by kindness and appreciation, which should be reflected in the robot’s tone, choice of words, and facial expressions if present. Participant feedback indicated that cultural customization of the robot’s appearance is a desired feature to make it more relatable, including the option to change eye colors, add a bindi, and modify clothing. Furthermore, introducing robot prototypes in local clinics as a preliminary step could promote familiarity and acceptance.

To address privacy concerns related to voice data collection, it is important to educate users, especially caregivers, about the robot’s support functions and limitations, what data is collected, and who has access. This requires transparent informed consent alongside compliance with established privacy and security standards, for example, the General Data Protection Regulation and Health Insurance Portability and Accountability Act. Technical safeguards, such as local storage or edge processing of sensitive data, and feature anonymization in voice analysis, can further reduce privacy risks inherent to speech processing.

Participants were informed of their right to withdraw from the study at any point. Future recruitment should further strengthen data governance by incorporating a consent clause that enables data withdrawal from protected institutional servers. In addition, future robot deployments should provide accessible training resources that help users understand the system’s capabilities and purpose, ultimately fostering long-term trust and acceptance. Such predeployment training should be tailored to differences in infrastructure and digital literacy between urban and rural Indian settings, which directly affect the feasibility of conversational AI implementation. Future deployments in India should consider the tradeoff between using local (on-device) models for speech recognition and synthesis and cloud-based architectures, such as those tested in this study. While the former reduces reliance on stable Wi-Fi connectivity, it operates with a more limited vocabulary and reduced contextual flexibility for verbal engagement. Adopting a hybrid design that combines local processing for essential interactions with cloud-based retrieval to sustain richer dialogue may provide a more robust and equitable pathway for conversational AI deployment across India’s diverse settings.

### Limitations and Future Work

Engaging people affected by dementia in research presents inherent challenges, and the requirement for fluency in English further limited the size and diversity of the participant cohort, as in India, more men from the current older generation acquired English as a second language. However, this study offered practical design considerations obtained from stakeholder feedback to enhance robot acceptance and the feasibility of implementation in India. Our study was designed to capture initial impressions and cultural perspectives from HRIs. Future longitudinal studies could address continuous interactions in home settings and the associated long-term implications.

Furthermore, future within-subject studies that systematically counterbalance the order of robot presentation could provide deeper insights into how specific robot features influence user acceptance. A future iteration of the work should implement the culturally tailored design features emerging from stakeholder feedback on robot prototypes for validation in real-world residential and clinical settings. In addition, as large language models continually change the landscape of open dialogue, integration with conversational robots represents a promising direction for engaging support among older adults [54]. Yet, potential risks, including the spread of misinformation or biases, should be addressed in the cultural feasibility exploration with stakeholder involvement.

Given the distinct impact of dementia on individuals, our findings cannot be generalized. However, we propose design considerations obtained from stakeholder perspectives on necessary improvements for sustained engagement when introducing robots into new cultural contexts. This includes incorporating features for local accent interpretation, customizing facial expressions, and implementing strategies to promote familiarity and accessibility.

### Conclusion

We pioneered a cultural feasibility study of conversational robots in India, an underrepresented context in AI and robotics for dementia care. We involved 29 stakeholders to evaluate human-robot engagement, robot acceptance, and perceptions regarding adopting the technology in India. Participants accepted and were willing to engage with conversational robots in verbal dialogue and recognized their potential to support daily routines, alleviate loneliness through personalized conversations, and promote cognitive engagement. Cultural adaptations to robot design and implementation are essential for feasibility in India, including enhanced speech processing for local accent interpretation and effective communication in noisy settings; a kind and appreciative communication style; customizable appearance through tone, choice of words, and facial expressions; and a phased introduction to promote familiarity with technology, starting in local clinics. This work paves the way for a more inclusive and culturally aware design of conversational robots for dementia care, particularly for feasible, real-world deployments and sustained user engagement in residential settings in India.

## Appendix

Multimedia Appendix 1 Supplementary materials: participant information, engagement and acceptance metrics, and interaction transcripts.

## Abbreviations

ADAS-Cog: Alzheimer’s Disease Assessment Scale-Cognitive Subscale
AI: artificial intelligence
CD: cognitive difficulty
CDR: Clinical Dementia Rating
HRI: human-robot interaction
OME: Observational Measure of Engagement
R: conversational social robot
RQ: research question
SAR: socially assistive robot
SCARF: Schizophrenia Research Foundation
