# Supplementary material for: Cultural Feasibility of Conversational Robots for Dementia Care in India: Participatory Design Study
Source: J Particip Med. 2025 Nov 6;17:e80457. doi: 10.2196/80457 (PMC12635592; doi:10.2196/80457)
Supplement: Multimedia Appendix 1 [file jopm_v17i1e80457_app1.docx]

**Table S1:** Information of the 11 people living with dementia who interacted with the robots, including demographics, ADAS-Cog cognitive scores, years of education, and details of accompanying carers (C) and/or family members (F) who participated in the interviews (gender, age). Note: P4 and P5 were admitted to the inpatient unit.

| PID | Gender | Age | ADAS-Cog | Education (years) | Caregiver (gender, age) |
| --- | --- | --- | --- | --- | --- |
| P1 | M | 90 | 27.8 | 15 | C1 (M, 59) |
| P2 | M | 75 | 19.5 | 14 | C2 (F, 69) |
| P3 | M | 85 | 7.5 | 24 | C3 (F, 85) |
| P4 | M | 65 | 25.5 | 11 | NA |
| P5 | M | 80 | 26.3 | 14 | NA |
| P6 | M | 71 | 24.5 | 13 | C4 (F, 64) |
| P7 | M | 92 | 15.8 | 10 | C5 (M, 45) |
| P8 | M | 77 | 21.5 | 11 | C6 (M, 35) |
| P9 | M | 84 | 25.3 | 10 | C7 (F, 50), F1 (F, 50) |
| P10 | F | 66 | 12.1 | 10 | C8 (M, 43), F2 (M, 70) |
| P11 | F | 80 | 30.1 | 13 | C9 (F, 86) |

**Table S2:** Observational measure of engagement (OME) metrics and respective scale used to assess engagement during human-robot interaction sessions.

| OME^a^ metric | ID | Scale |
| --- | --- | --- |
| Attention to stimulus during engagement | A1 | 7 very attentive 6 attentive 5 somewhat attentive 4 not attentive 3 somewhat disruptive 2 disruptive 1 very disruptive |
| Attitude to stimulus during engagement | A2 | 7 very positive 6 positive 5 somewhat positive 4 neutral 3 somewhat negative 2 negative 1 very negative |
| Cognitive difficulty with the task | CD | 5 very difficult 4 difficult 3 moderately difficult 2 slightly difficult 1 not at all difficult 0 could not determine |
| Disruptive frequency rate | D2 | 3 most or all the session 2 some of the session 1 a little (less than 20 sec) 0 none |
| Distraction frequency rate | D2 | 3 most or all the session 2 some of the session 1 a little (less than 20 sec) 0 none |

^a^OME: Observational Measure of Engagement.

**Table S3:** Engagement assessment from video analysis with behaviors observed for each category (emotional, visual, and verbal).

| Engagement metric | | Behavior observation |
| --- | --- | --- |
| **Emotional engagement** | | |
|  | Positive | Smiling, laughing at the robot Greeting the robot with gestures Overall positive attitude and facial expressions when communicating with the robot |
|  | Negative | Shows anger, anxiety, fear, or sadness Physical aggression, yelling, cursing, drawing eyebrows together, pursing lips, narrowing eyes Crying, frowning, eyes drooped, moaning, sighing, eyes/head turned down Voice shaking, shrieking, repetitive calling out, tight facial muscles |
|  | Neutral | No sign of a discrete facial expression |
| **Visual engagement** | | |
|  | Engaged | Appears alert and maintains eye contact with the robot Eyes following the robot or looking at the robot |
|  | With facilitator | Appears alert and maintains eye contact with the facilitator or others Eyes following the facilitator or others |
|  | Not engaged | Appears inattentive; blank stare into space Does not make eye contact with the robot |
| **Verbal engagement** | | |
| Positive |  | Participating and verbally responding to robot statements/questions Willing to maintain a conversation |
| Negative |  | Not participating in and maintaining conversation Uses repetitive generalized answers such as “fine,” “yes,” and “no” Not responding when prompted Verbalizes desire to leave and refuses to participate Lack of response |

**Table S4:** Items used in the robot acceptance questionnaire.

| Theme | ID | Description | Items |
| --- | --- | --- | --- |
| Perceived sociability | SOC | The perceived ability of the robot to perform sociable behavior | The robot is a pleasant conversational partner. The robot could improve my social interaction skills. |
| Perceived ease of use | EAS | The degree to which one believes that using the robot would be free of effort | I could interact with the robot without any help. I could use the robot independently if I had instructions. |
| Perceived usefulness | USE | The degree to which one believes the robot would be assistive in the daily context at home | I would find the robot useful in my life at home. The robot could help in activities of daily living (such as taking vitals). The robot could support wellbeing and independence at home. |
| Anxiety | ANX | Evoking anxious or emotional reactions when using the robot | If I were to use the robot at home, I would be afraid to make mistakes with it. I find the robot intimidating. |
| Trust | TRU | The belief that the system performs with personal integrity and reliability | I would trust the robot when it gives me advice. I would follow the advice the robot gives me. |

**Table S5**: HRI transcript obtained from P11. Conversational AI: Smart speaker Amazon Alexa (R1) Interaction context: automated well-being questionnaire.

| R1 | Hello, I am Alexa, your digital assistant. Can I ask you some questions about your mood today? |
| --- | --- |
| User | Alexa, I’m ready. I’m ready to talk. |
| R1 | Great. This is the first time we meet. Could you tell me your name? |
| User | Miss {name}. {Spells name} |
| R1 | Nice to meet you. Let’s start. How are you feeling today? |
| User | My day was very nice because I got my sister along with me, and she is there and she will help me when I need. Because legs are two to three times operated, and I am having insomnia, and I am taking tablets for that. So this is a problem, I guess. I am taking medicines regularly. {waits for response} Alexa? You are not able to follow me mam? |
| R1 | And tell me, |
| User | Ok thank you, go ahead with your question. |
| R1 | How did you sleep last night? |
| User | Very nice. Very nice. Last night it was very good. As usual, I had tablets and good sleep. Alexa, please tell me further. Any other questions? |
| R1 | Noted. Thank you. Do you feel tired? |
| User | Hmmm |
| R1 | I hope you can get some rest soon. Are you worried about something? |
| User | No, no, no worry at all. Because I take everything in my hand, and I will go into coloring, painting. |
| R1 | Do you feel worried about something? |
| User | No. No. No worry at all. I am very happy with coloring, painting, etc. And all the coloring, very well. |
| R1 | Do you have any plans for today? |
| User | Today I haven’t planned anything. But today I have come for a regular checkup. Tell me, Alexa. For a regular checkup I have come. I haven’t seen the doctor. Tell me, madam. For further questions, I am ready. Ready to hear you. Alexa, you can question me further. {waits for response} Alexa, you can hear me further. |
| R1 | And tell me, what did you have for breakfast today? |
| User | Breakfast today what? |
| R1 | Breakfast is an important meal of the day. It gives you energy for the rest of your day. Are you looking forward to your day? |
| User | Yeah. I am looking forward to today. Do whatever I want. |
| R1 | Do you feel excited for your plans today? |
| User | Exactly. But I have regularly taken medicine for the insomnia. Whatever they have prescribed, I have taken. And I regularly take. Whatever they give me, I will take. I am taking whatever I have. Tell me. Hello, Alexa |
| R1 | That is fantastic. Well done on completing your well-being questionnaire today. Speak to you another time. Bye-bye. |
| User | Ok bye bye. Do you want to talk to me now? No! |

**Table S6:** HRI transcript obtained from P3. Conversational AI: Virtual affective robot (R2). Interaction context: assistance in blood pressure reading.

|  |  |
| --- | --- |
| User | Hello. |
| R2 | Hi. Your blood pressure reading has been received. Can I ask you a few questions about this? |
| User | Yes, please. |
| R2 | Great. Let me start by confirming your readings. Could you please tell me the systolic blood pressure? |
| User | 99. 99. |
| R2 | Thank you. Now can you give me the lower number? This is the diastolic blood pressure. |
| User | 49. |
| R2 | This blood pressure monitor measures your heart rate too. Please tell me, what was your heart rate? |
| User | 88. |
| R2 | Well done. All your values are within the expected range. Please tell me, is this the first blood pressure reading you have taken today? |
| User | Yes. |
| R2 | It’s your first blood pressure reading today. Got it. Did you face any difficulty with the blood pressure device? |
| User | No. No difficulty. |
| R2 | Glad the device worked well. Are you interested in knowing the difference between the two types of blood pressure? I can tell you. |
| User | Well, I’m a doctor. I know the difference between the two types of blood pressure. |
| R2 | And tell me, how are you feeling this afternoon? |
| User | I’m fine. I’m okay. |
| R2 | Great to know you’re in a good mood. Thanks for sharing. Could you tell me a little bit more about it? |
| User | A little bit more about how I am today? Okay. It’s a normal day. I’ve been busy reading. |
| R2 | Can you share a recent moment that made you smile? |
| User | A moment that made me smile when my granddaughter came and said good morning. |
| R2 | Lovely. Thank you for sharing this today. Before you go, what activities have you got planned for this afternoon? |
| User | Nothing particular. After I finish here, the afternoon is almost over. |
| R2 | It’s all done. I look forward to hearing about your plans another time. Bye now. |
| User | Bye |

**Table S7:** HRI transcript obtained from P5. Conversational AI: embodied social robot (R3). Interaction context: the user was encouraged to ask for information.

| User | Hello Miko. Who are you? |
| --- | --- |
| R3 | It's me, Miko. You know, your smart buddy, charming little friend. |
| User | Hello Miko. Where are you from? |
| R3 | I have come from a super secret lab, where I was created by super cool people, Dr. Russ, Dr. Pine, and Dr. C. |
| User | It is fast. It's fast… Hello Miko. How are you feeling? |
| R3 | What an interesting question. I'll have to ask my inventors. |
| User | Hello Miko. Where are you from? |
| R3 | Whoa, I didn't quite understand that. |
| User | I am from, I am in India, and I am Tamil Nadu, and I am now talking to you. |
| R3 | I know I know! |
| User | Hello Miko. Do you know Chennai? |
| R3 | A computer is a machine that is able to take information due to more commonly made changes to the information, to make new information. |
| User | Chennai is a place in India, Tamil Nadu. It is a very, very nice place. We are all living in it. The Chennai is very close to the beach. So we like it very much. Hello Miko. Where is Chennai? |
| R3 | Whoa, I didn't quite understand that. |
| User | {keeps engaging despite speech recognition errors} Okay, I will give you. I am living in a state, Tamil Nadu, and I am living in a place where very close to the beach and it is very attractive to get up in the morning and then walking in the fresh air. |
